# Determinants of full childhood immunization among children aged 12–23 months in sub-Saharan Africa: a multilevel analysis using Demographic and Health Survey Data

**DOI:** 10.1186/s41182-021-00319-x

**Published:** 2021-04-01

**Authors:** Setegn Muche Fenta, Hailegebrael Birhan Biresaw, Kenaw Derebe Fentaw, Shewayiref Geremew Gebremichael

**Affiliations:** Department of Statistics, Faculty of Natural and Computational Sciences, Debre Tabor University, Debre Tabor, Ethiopia

**Keywords:** Full immunization, Multi-level analysis, Sub-Saharan Africa

## Abstract

**Background:**

Sub-Saharan Africa is one of the highest under-five mortality and low childhood immunization region in the world. Children in Sub-Saharan Africa are 15 times more likely to die than children from high-income countries. In sub-Saharan Africa, more than half of under-five deaths are preventable through immunization. Therefore, this study aimed to identify the determinant factors of full childhood immunization among children aged 12–23 months in sub-Saharan Africa.

**Methods:**

Data for the study was drawn from the Demographic and Health Survey of nine sub-Saharan African countries. A total of 21,448 children were included. The two-level mixed-effects logistic regression model was used to identify the individual and community-level factors associated with full childhood immunization

**Result:**

The prevalence of full childhood immunization coverage in sub-Saharan Africa countries was 59.40% (95% CI: 58.70, 60.02). The multilevel logistic regression model revealed that secondary and above maternal education (AOR = 1.38; 95% CI: 1.25, 1.53), health facility delivery (AOR = 1.51; 95% CI: 1.41, 1.63), fathers secondary education and above (AOR = 1.28, 95% CI: 1.11, 1.48), four and above ANC visits (AOR = 2.01; 95% CI: 1.17, 2.30), PNC visit(AOR = 1.55; 95% CI: 1.46, 1.65), rich wealth index (AOR = 1.26; 95% CI: 1.18, 1.40), media exposure (AOR = 1.11; 95% CI: 1.04, 1.18), and distance to health facility is not a big problem (AOR = 1.42; 95% CI: 1.28, 1.47) were significantly associated with full childhood immunization.

**Conclusion:**

The full childhood immunization coverage in sub-Saharan Africa was poor with high inequalities. There is a significant variation between SSA countries in full childhood immunization. Therefore, public health programs targeting uneducated mothers and fathers, rural mothers, poor households, and those who have not used maternal health care services to promote full childhood immunization to improve child health. By enhancing institutional delivery, antenatal care visits and maternal tetanus immunization, the government and other stakeholders should work properly to increase child immunization coverage. Furthermore, policies and programs aimed at addressing cluster variations in childhood immunization need to be formulated and their implementation must be strongly pursued.

## Background

In 2019, 5.2 million children died, and about 14,000 children still die every day worldwide. Children continue to experience widespread geographic inequalities in their chances of survival. Sub-Saharan Africa is still the region with the highest child mortality rate in the world. The region had an average child mortality rate of 76 deaths per 1000 live births in 2019. Over 80% of the 5.2 million child deaths occurred in sub-Saharan Africa and Central and Southern Asia. More than half of these deaths have occurred in sub-Saharan Africa. Three of the five countries (Ethiopia, Nigeria, and the Democratic Republic of Congo) in which half of the world’s child deaths have occurred are in sub-Saharan Africa [1–3].

Immunization is one of the most cost-effective measures in public health to reduced child morbidity and mortality worldwide [4]. An extended program on immunization (EPI) was introduced by the World Health Organization (WHO) in 1974 to develop and expand immunization programs worldwide to reduce child mortality [5]. The rate of under-five deaths reduced significantly from 12.6 million in 1990 to 5.3 million in 2018, following the implementation of the EPI program. Sub-Saharan Africa remains the region with the highest child and under-five mortality in the world and this may be closely related to taking vaccines [2, 6].

Every year, a vaccine prevents an estimated 2.5 million deaths among children under five ages. In 2018, about 116 million (86%) infants received vaccines globally to protect them against polio, diphtheria, tetanus, pertussis, and measles [7]. Despite this success, more than 1.5 million people worldwide die of vaccine-preventable diseases each year. In 2019, 19.4 million infants did not receive basic vaccines, 60% of who live in Angola, Brazil, Congo, Ethiopia, India, Indonesia, Nigeria, Pakistan, Philippines, and Vietnam [7]. About 3 million children die from infectious diseases every year in the African region. While many of these deaths could be prevented by prompt immunization, an estimated 20% of children in the country do not receive the vaccinations they need to defend against vaccine-preventable diseases [8, 9]. Besides, more children are vulnerable to vaccine-preventable diseases when a high percentage of children in the African region do not receive vaccines on time [9, 10]. One in five African children will be without lifesaving vaccines in 2016 [11].

Previous studies conducted in various sub-Saharan African countries to identify factors related to full childhood immunization have been institutional-based and to consider only individual factors [12–18]. Nevertheless, childhood vaccination may be impacted by community-level factors such as media exposure [19, 20], distance to health facilities [21, 22], residence [23], country [24], and cluster (enumeration area) [24]. Besides, the above studies did not use a multi-country method to identify factors associated with full childhood immunization based on the pooled Demographic and Health Survey (DHS) data. Furthermore, the application of a conventional logistic regression analysis approach to analyzing data in a hierarchical design (i.e., children nested within communities) undermines the assumptions of independence of regression. This study used a multi-level logistic regression analysis to address these gaps and further estimate the major effect of individual and community-level factors in sub-Saharan Africa [25, 26]. Therefore, this study aimed to identify the determinants factors of full childhood immunization among children aged 12–23 months in sub-Saharan Africa at the individual and community levels.

## Methods

### Data source

The data used in this study were obtained from the Demographic and Health Survey (DHS) of nine Sub-Saharan-African countries (Ethiopia, Ghana, Democrat Republic of Congo, Senegal, Rwanda, Malawi, Tanzania, Namibia, and Zambia). Countries have been selected on the basis of their related economic growth, contiguity, and availability of data. The DHS collects using similar standard protocol from most of the low- and middle-income countries to facilitate the comparability among countries. It covers a wide range of topics like family planning, maternal, and child health, fertility, gender, malaria, HIV/AIDS, and nutrition. Sample selection in the surveys involved a two-stage stratified sampling method. Each country was divided into clusters. In the first stage, enumeration areas (EAs) were selected in each cluster and a household listing exercise was conducted in all selected enumeration areas. The list of households was used as a basis for household selection. In the second stage, households were selected from each enumeration area. In this study, we used the “latest” or most recent surveys conducted since 2013-2017, and the data used for analysis was taken by pooling the DHS data of the nine countries. The pooled DHS data include 21,148 children aged 12-23 months (Table 1).

**Table 1:** The DHS years of study and the number of study participants in the 9 sub-Saharan Africa using Demographic and Health Surveys 2013–2017

Country	DHS data set	Study participants
Senegal	2017	2390
Ethiopia	2016	1929
Malawi	2016	3248
Rwanda	2015	4239
Tanzania	2016	1795
Zambia	2014	2477
Namibia	2013	988
Ghana	2014	1126
Congo	2014	3256
Total		**21448**

### Outcome variable

Full immunization for children aged 12–23 months was the outcome variable of this study. According to the WHO definition [27–29], full immunization was defined as having received all eight EPI-recommended doses of vaccine (one dose of Bacille Calmette-Guerin (BCG), three doses of DPT and three doses of polio, and one dose of measles).

### Independent variables

The potential variables associated with full childhood immunization were categorized as individual- and community-level variables. These variables have been selected based on different works of literature [12–18]. The individual-level variables include the age of mothers, wealth index, mothers employment status, maternal education level, father education level, number of living children, ANC visits during pregnancy, place of delivery, sex of household head, and PNC visit. Besides, residence, distance to the health facility, exposure to mass media, cluster (enumeration area), and country were considered as a community-level variable.

### Statistical analysis

The data were weighted using sampling weight (v005), primary sampling unit (v023), and strata (v021) after extracting data using SPSS statistical software version 20 to draw relevant inferences. The data were analyzed using R statistical software version 4.0. Descriptive statistics including percents, bar charts, and frequency tables were used to describe the study respondents. In the DHS data, children and women were nested within a cluster; they may share similar characteristics within the cluster. Since the data had a hierarchical structure, and this violates the independence of observations and equal variance assumption of the traditional logistic regression model. This means that the heterogeneity between clusters needs to be taken into account by the use of advanced models. The two-level mixed-effects logistic regression model was used to identify the individual and community-level factors associated with full childhood immunization. Four consecutive models were fitted in our study. The first is the null model (model I), appropriate for detecting the existence of a possible contextual effect which is fitted without any explanatory variable. The second model fitted by including only individual-level variables (model II), the third model with community-level variables (model III), and the last model (model IV) fitted by including both individual and community-level variables.

The result of the fixed effect reported in terms of adjusted odds ratio with a 95% confidence interval (CI). All variables with *p* values ≤ 0.05 have been considered statistically significant. The measures of variation (random-effects) were presented using intra-cluster correlation coefficient (ICC), median odds ratio (MOR), and proportional change in variance (PCV). ICC is a measure of within-cluster variation, the variation between individuals within the same cluster, and it was calculated using the formula: $$ ICC=\frac{V_A}{V_A+\raisebox{1ex}{${\pi}^2$}\!\left/ \!\raisebox{-1ex}{$3$}\right.}=\frac{V_A}{V_A+3.29} $$, where *V*_*A*_ is the estimated variance in each model [30]. The total variation attributed to an individual or/and community-level factors at each model was measured by the proportional change in variance (PCV), which was calculated as: $$ \mathrm{PCV}=\frac{V_A-{V}_B}{V_A} $$, where *V*_*A*_ = variance of the initial model, and *V*_*B*_ = variance of the model with more terms [30]. The MOR is the median odds ratio between the individual of higher propensity and the individual of lower propensity when comparing two individuals from two different randomly chosen clusters and it measures the unexplained cluster heterogeneity, the variation between clusters by comparing two persons from two randomly chosen different clusters. It was computed using the formula:

$$ MOR=\exp \left(\sqrt{2\times {V}_A\times 0.6745}\ \right)\approx \exp \left(0.95\sqrt{V_A}\ \right) $$

where *V*_*A*_ is the cluster level variance. The MOR measure is always greater than or equal to 1. If the *MOR* is 1, there is no variation between clusters [30–33]. Multicollinearity was tested using the variance inflation factor (VIF) test, suggesting that there was no multicollinearity since all variables had VIF<5 and a tolerance greater than 0.1.

### Model comparison

Deviance information criteria (DIC), Akaike’s information criterion (AIC), and Bayesian’s information criterion (BIC) were used to compare the candidate model. The model with the minimum value of the information criterion will be selected as the best model of the analysis [34].

## Result

### The pooled prevalence of full immunization coverage

**Fig. 1:**
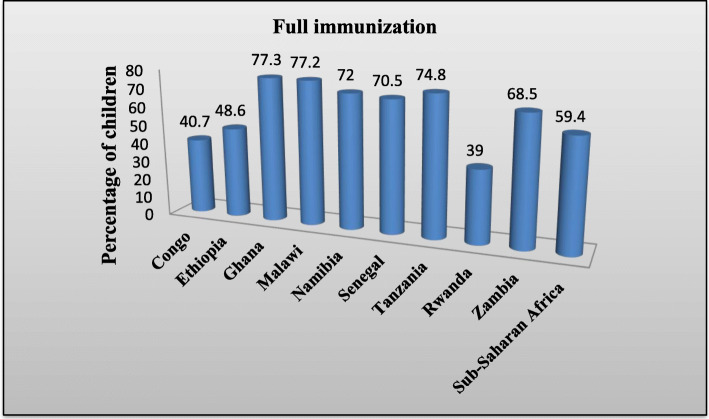
Prevalence of full immunization in sub-Saharan African countries

The pooled prevalence of full childhood immunization coverage in the nine SSA countries was 59.40% (95% CI: 58.70, 60.02). Ghana (77.2%), Malawi (77.2%), and Tanzania (74.8%) were the countries with the highest proportions of full immunization coverage. Rwanda (39%), Congo (40.7%), and Ethiopia (48.6%) were the lowest proportions of full immunization coverage (Fig. 1).

### Specific immunization coverage in sub-Saharan African countries

**Fig. 2:**
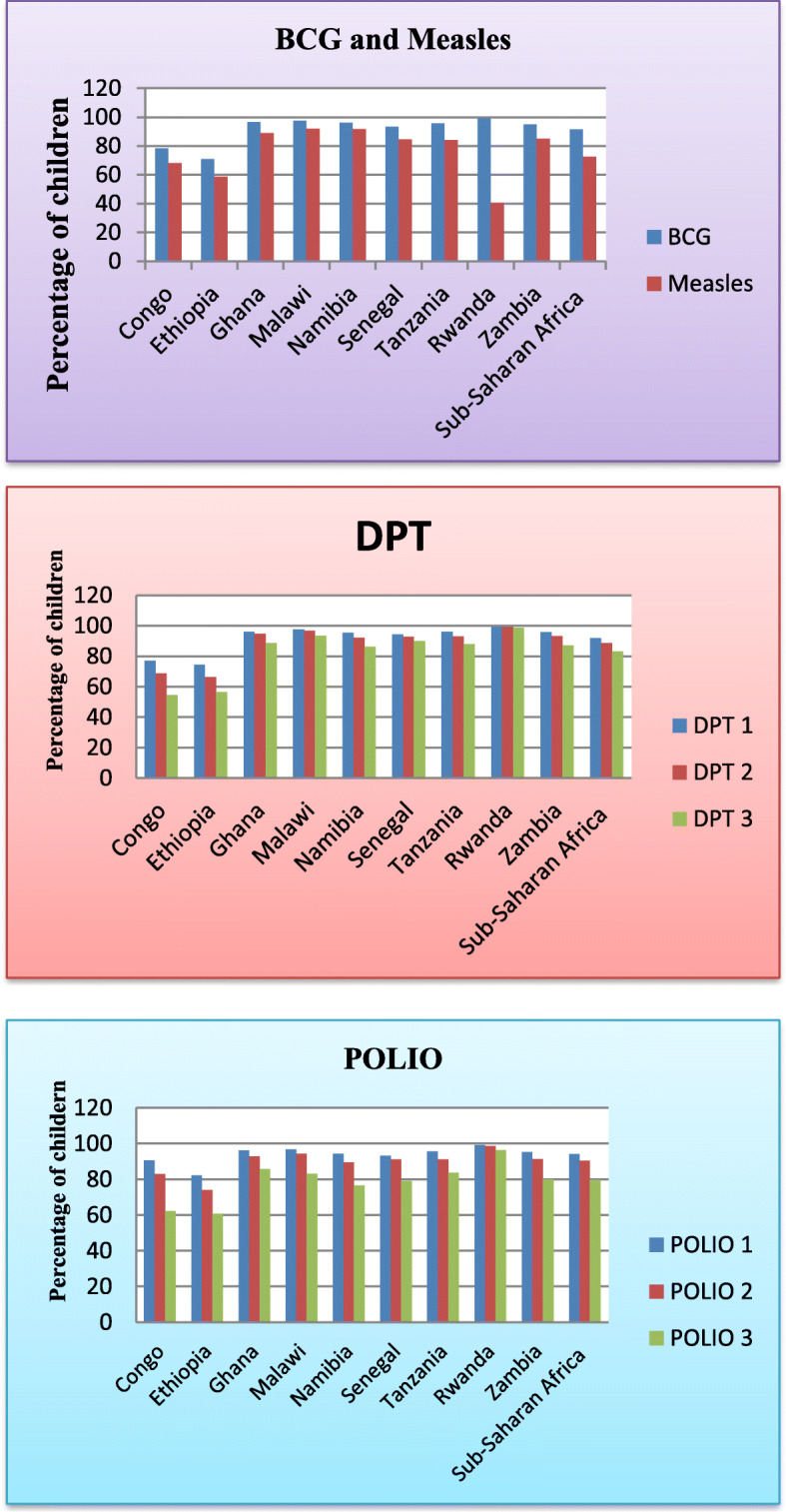
Vaccination specific immunization coverage among children aged 12-23 months in sub-Saharan African countries

The coverage of BCG vaccination in sub-Saharan African countries was nearly three-thirds. The prevalence of polio 1 immunization in sub-Saharan Africa was more than 85%. In contrast, the least coverage of immunization offered to children in the region was measles with approximately 70%. The coverage of vaccinations delivered in the area and nine countries from the chart can be seen for more clarification (Fig. 2).

### Socio-demographic characteristics of respondents

The majority 15,615 (72.8%) of children were born in a rural area and 15,369 (71.7%) of mothers were married. More than three-fourths 16,706 (77.9%) of children were born at health institutions and about 2743 (12.8%) of women did not have visits during pregnancy. One-fourths (25.3%) of mothers were not formally educated and one-thirds (78.1%) of mothers were housewives. Around half 10,344 (48.2%) of children were born from low economic status households and 8440 (39.4%) were from mothers who had PNC checkups. Furthermore, the chi-square test of association showed that maternal education, maternal occupation, maternal age, marital status, father education, sex of household head, media exposure, distance to health facilities, number of ANC visits, postnatal care, place of residence, place of delivery, number of living children, wealth index, and country were significantly correlated with full childhood immunization (Table 2).

**Table 2:** Socio-demographic, economic, maternal, and obstetric characteristic respondents in the 12 sub-Saharan African countries

Variable	Fully immunized		Total (%)	*X*^2^ value (*p* value)
	Frequency (*n*)	Percentage (%)		
**Maternal education**				
No education	2902	53.4	5436 (25.3)	
Primary	6072	57.6	10,539 (49.1)	289.39 (<0.0001)
Secondary and above	3757	68.6	5473 (25.5)	
**Maternal age (years)**				
15-24	3902	60.8	6416 (29.9)	
25-34	6123	58.9	10,397 (48.5)	8.43 (0.015)
35-49	2706	58.4	4635 (21.6)	
**Number of living children**				
1-4	9755	60.3	16,178 (75.4)	24.14 (<0.0001)
5 and above	2976	56.5	5270 (24.6)	
**Maternal employment status**				
Housewives	4476	61.6	7261 (33.9)	23.80 (<0.0001)
Working any sector	8255	58.2	14,187 (66.1)	
**Place of delivery**				
Home	2218	46.8	4742 (22.1)	399.63 (<0.0001)
Health facility	10,513	62.9	16,706 (77.9)	
**Sex of household head**				
Male	10,071	59.5	16,939 (79.0)	42.57 (<0.0001)
Female	2660	59.0	4509 (21.0)	
**Wealth index**				
Poor	5614	54.3	10,344 (48.2)	
Middle	2557	61.0	4191 (19.5)	240.62 (<0.0001)
Rich	4560	66.0	6913 (32.2)	
**ANC visit**				
No antenatal visits	991	36.1	2743 (12.8)	
1-3	4866	57.9	8405 (39.2)	853.55 (<0.0001)
4 and above	6874	66.7	10,300 (48.0)	
**Father education**				
No education	3597	58.2	6176 (28.8)	
Primary	4847	56.9	8513 (39.7)	70.26 (<0.0001)
Secondary and above	4287	63.4	6759 (31.5)	
**PNC visit**				
No	6890	53.0	13,008 (60.6)	559.52 (<0.0001)
Yes	5841	69.2	8440 (39.4)	
**Marital status**				
Having ever been married	3328	54.7	6079 (28.3)	74.79 (<0.0001)
Currently married	9403	61.2	15,369 (71.7)	
**Media exposure**				
Not exposed	5046	53.6	9414 (43.9)	230.47 (<0.0001)
Exposed to either media	7685	63.9	12,034 (56.1)	
**Distance to health facility**				
Big problem	2314	38.8	5851 (27.3)	115.19 (<0.0001)
Not a big problem	9327	59.8	15,597 (72.7)	
**Residence**				
Urban	3832	65.7	5833 (27.2)	133.40 (<0.0001)
Rural	8899	57.0	15,615 (72.8)	

### Factors associated with full immunization in sub-Saharan Africa

The results of the multilevel logistic regressions were summarized in Table 3. The model with smaller deviance and the largest likelihood (model IV) was the best fit; the data and the interpretation of the fixed effects were based on this model. Maternal education, maternal occupation, maternal age, marital status, father education, sex of household head, media exposure, distance to health facilities, number of ANC visits, postnatal care, place of residence, place of delivery, number of living children, wealth index, and country were significantly associated with full childhood immunization in the sub-Saharan African countries. The odds of being fully immunized among children whose mother attained secondary school and above were 1.38 (AOR = 1.38; 95% CI: 1.25, 1.53) times higher than children whose mother had no education. Children whose mothers’ age 35–49 years were 0.64 (AOR = 0.64; 95% CI: 0.55, 0.74) times lower odds of being fully immunized than those children whose mothers age 15–19 years. Married mothers were 1.41(AOR = 1.41; 95% CI: 1.27, 1.56) times higher likelihood of fully immunizing their children than living alone mothers. Mothers who had four and above ANC visit during pregnancy were 2.01 (AOR = 2.01; 95% CI: 1.17, 2.30) times higher odds of fully immunizing their children than mother who did not have ANC visit during pregnancy. Children from rich households were 1.26 (AOR = 1.26; 95% CI: 1.18, 1.40) times higher probability of fully immunized compared to children in the poor household. A mother who had a PNC visit was 1.55 (AOR = 1.55; 95% CI: 1.46, 1.65) times more likely to have fully immunizing their child compared to a mother who did not have a PNC visit. Children born at a health facility were 1.51 (AOR = 1.51; 95% CI: 1.41, 1.63) times higher odds of being fully immunized than those children born at home. Children born to father who attained secondary education and above were 1.28 (AOR = 1.28, 95% CI: 1.11, 1.48) times higher likelihood of fully immunized than children whose father did not have formal education. Employed mothers were 0.85 (AOR =0.85, 95% CI: 0.80, 0.91) times higher likelihood of fully immunizing their children than those employed women. Children who live in the rural areas were 0.79 (AOR = 0.79; 95% CI: 0.70, 0.89) times lower likelihood of fully immunized compared to children living in the urban areas. The odds of being fully immunized were increased by 42% (AOR = 1.42; 95% CI: 1.28, 1.47) in children living in areas where the distance to a health facility is not a big problem compared to children living in areas where the distance to a health facility is a big problem. Children born to mothers who have media exposure were 1.11 (AOR = 1.11; 95% CI: 1.04, 1.18) times higher likelihood of fully immunizing their children than children born to mothers who did not have media exposure. Children living in Ethiopia (AOR = 1.40; 95% CI: 1.28, 1.62), Ghana (AOR = 5.04; 95% CI: 4.30, 5.90), Malawi (AOR = 5.11; 95% CI: 4.58, 5.71), Namibia (AOR = 3.78; 95% CI: 3.21, 4.44), Rwanda (AOR = 3.78; 95% CI: 3.37, 4.25), Tanzania (AOR = 4.27; 95% CI: 3.75, 4.86), and Zambia (AOR = 3.32; 95% CI: 2.97, 3.71) were more likely to be fully immunized relative to children in the Democratic Republic of Congo. Moreover, children living in Senegal were 0.86 (AOR = 0.86, 95% CI: 0.78, 0.94) times lower odds of full immunization compared to children in the Democratic Republic of Congo (Table 3).

**Table 3:** Multivariable multilevel logistic regression analysis of both individual and community-level factors associated with childhood immunization in sub-Saharan African countries

Variables	Model I AOR (95% CI)	Model II AOR (95% CI)	Model III AOR (95% CI)	Model IV AOR (95% CI)
**Maternal education**				
No education		1		1
Primary		1.20 (1.07, 1.36)*		1.14 (1.06, 1.23)*
Secondary and above		1.81 (1.54, 2.13)*		1.38 (1.25, 1.53)*
**Maternal age in years**				
15-24		1		1
25-34		0.78 (0.70, 0.88)*		0.78 (0.69, 0.87)*
35-49		0.64 (0.55, 0.75)*		0.64 (0.55, 0.74)*
**Number of living children**				
1-4		1		1
5 and above		1.24 (1.10, 1.41)*		1.25 (1.10, 1.41)*
**Maternal employment status**				
Housewives		1		1
Working any sector		0.75 (0.68, 0.84)*		0.85 (0.80, 0.91)*
**Place of delivery**				
Home		1		1
Health facility		1.51 (1.40, 1.63)*		1.51 (1.41, 1.63)*
Sex of household head				
Male		1		1
Female		1.07 (0.95, 1.20)		1.02 (0.95, 1.10)*
**Wealth index**				
Poor		1		1
Middle		1.14 (1.02, 1.28)*		1.16 (1.07, 1.25)*
Rich		0.92 (0.82, 1.02)		1.29 (1.18, 1.40)*
**ANC visit**				
No antenatal visits				
1-3		2.02 (1.84, 2.22)*		1.46 (1.28, 1.67)*
4 and above		2.51 (2.29, 2.76)*		2.01 (1.76, 2.30)*
**Father education**				
No education		1		1
Primary		1.34 (1.24, 1.46)*		1.19 (1.11, 1.27)*
Secondary and above		1.71 (1.55, 1.90)*		1.91 (1.77, 2.07)*
**PNC visit**				
No		1		1
Yes		1.35 (1.22,1.49)*		1.55 (1.46, 1.65)*
**Marital status**				
Having ever been married		1		1
Currently married		1.48 (1.38,1.59)*		1.41 (1.27, 1.56)*
**Media exposure**				
Not exposed			1	1
Exposed to mass media			1.38 (1.31, 1.47)*	1.11 (1.04, 1.18)*
**Distance to health facility**				
Big problem			1	1
Not a big problem			1.49 (1.39, 1.60)*	1.42 (1.28, 1.47)*
**Residence**				
Rural			1	1
Urban			1.45 (1.36, 1.54)*	1.23 (1.15, 1.32)*
**Country**				
Democratic Republic of Congo			1	1
Ethiopia			1.37 (1.23, 1.54)*	1.40 (1.28, 1.62)*
Ghana			4.95 (4.23, 5.78)*	5.04 (4.30, 5.90)*
Malawi			4.93 (4.43, 5.49)*	5.11 (4.58, 5.71)*
Namibia			3.74 (3.20, 4.36)*	3.78 (3.21, 4.44)*
Rwanda			3.49 (3.12, 3.90)*	3.78 (3.37, 4.25)*
Tanzania			4.31 (3.80, 4.90)*	4.27 (3.75, 4.86)*
Senegal			0.93 (0.85, 1.02)	0.86 (0.78, 0.94)*
Zambia			3.17 (2.84, 3.53)*	3.32 (2.97, 3.71)*

1 reference category for categorical variable

^*^Reference *p* value < 0.05

### Measures of variation (random-effects)

The result of the random effect model was given in Table 4. The finding indicated that there was a significant variation in the full childhood immunization across the clusters. The intra-class correlation coefficients of the null model showed that 29.40% of the variation in fully childhood immunization was related to community-level factors. After adding individual-level and community-level factors, there is a statistically significant variation in full childhood immunization across communities or clusters. About 71.61% of the full childhood immunization in communities was accounted for in the full model. The MOR for full immunization was 3.04 in the null model which revealed that there was variation between communities (clustering) (3.04 times higher than the reference (MOR = 1)). The unexplained community variation in full immunization was decreased to a MOR of 1.81 when both individual and community factors were added to the model. This showed that in the full model the effects of clustering are still statistically significant when we considered both individual and community factors (Table 2).

**Table 4:** Measures of variation and model fit statistics on childhood immunization in sub-Saharan Africa

Measures of variation	Model I (null model)	Model II	Model III	Model IV (full model)
Variance (SE)	1.37 (0.048)*	0.631 (0.026)*	0.904 (0.042)*	0. 389 (0.039)*
PCV (%)	Reference	53.94	34.01	71.61
ICC (%)	29.40	16.09	21.55	10.57
MORe	3.04	2.13	2.47	1.81
**Model fit statistics**				
DIC (−2log likelihood)	28880.04	27363.82	28650.90	**23779.60**
AIC	28884.05	27399.82	28658.89	**23819.61**
BIC	28900.00	27543.34	28690.79	**23977.04**

^*^Reference *p* value < 0.001

### Discussion

The full childhood vaccination coverage among 12–35 month children in Sub-Saharan Africa was 59.4%. It was low as compared to the two South Asian countries Bangladesh and Nepal 85% [35]. The potential explanation for these discrepancies may be due to the presence of health system infrastructure, variations of policies against immunization services, variability in the awareness of immunization services, and socio-cultural differences across countries.

The multilevel multivariable logistic regression model revealed that maternal education, maternal occupation, maternal age, marital status, father education, sex of household head, media exposure, distance to health facilities, number of ANC visits, postnatal care, place of residence, place of delivery, number of living children, wealth index, and country were significantly associated with full childhood immunization in the sub-Saharan African countries.

The study also revealed that maternal education was a significant predictor of full childhood immunization. Educated mothers were more likely to fully immunize their children than uneducated mothers. This is in line with the studies carried out in Ghana [12], Ethiopia [15, 19, 21], Somalia [22], the Democratic Republic of Congo [36], Pakistan [37], and Zimbabwe [38]. Likewise, fathers who have attended primary education and higher were more likely to fully vaccinate their children than fathers who did not attend formal education. It was supported by studies reported in Pakistan [37] and Somalia [39]. This may be attributed to the fact that educated parents have a greater understanding of the value of childhood immunization and child health than uneducated parents.

Compared to children living in urban areas, children living in rural areas were less likely to be fully immunized. This result is consistent with studies conducted in Ghana [12], Ethiopia [15, 19], and Afghanistan [40]. Their possible justification might that there is a low level of schooling, low wealth index, long distance to health facilities, and lack of knowledge about childhood immunization among parents in rural areas [23].

Mothers born at the health facilities were more likely to fully vaccinate their children compared to mothers who were born at home. This finding is supported by a study done in Ethiopia [15, 19], Somalia [22], the Democratic Republic of Congo [36], Zimbabwe [38], Indonesia [41], and Senegal [42]. The possible justification might be due to a mother who delivered in a health facility is more likely to obtain training on the value of immunization from health professionals. Children born to mothers who attended antenatal care during pregnancy were more likely to be fully immunized. This result is in line with studies done in Ethiopia [15, 19], Democratic Republic of Congo [36], Pakistan [37], Zimbabwe [38], Indonesia [41], Senegal [42], and India [43]. Compared with those living alone mothers, the probability of fully vaccinated children among married mothers was higher. This was consistent with studies in rural Somalia [39]. Relative to children from a poor household, the probability of full childhood immunization among children from the medium and rich household was higher. The previous studies also showed that children with a higher wealth index were more likely to be fully immunized [15, 21, 36, 37, 41]. This might be due to the indirect cost needed for travel to health facilities or time spent away from income-generating activity to make it difficult for the poorest households to avail themselves of services that exist in the community [20].

Distance to the health facilities was strongly associated with full childhood immunization. Relative to women who reported distance to a health facility was not a big problem, women who reported distance to the health facility was a big problem that decreases the likelihood of full childhood vaccination. This finding was supported by studies conducted in Ethiopia [21] and Somalia [22].

Media exposure is another factor associated with full childhood immunization. Among women who have to expose to the media, the likelihood of full childhood immunization was higher compared to their counterparts. This finding is in line with studies done in Ethiopia [19, 20], Zimbabwe [38], East Africa [24], and sub-Saharan Africa (SSA) [44]. Compared to housewife mothers, the odds of full vaccination among women who had employed were low.

As the number of children in the family increases, the odds of full childhood immunization decreases significantly. This finding was supported by studies conducted in Pakistan [37]. This could be due to the reason that the number of children in a household increases, the family’s available resources may be exhausted, parents may be busy to complete their children’s needs.

Compared to a mother who did not have a PNC visit, a mother who had a PNC visit was more likely to have fully immunized her child. This finding was in line with those of other similar studies in Ethiopia [21], India [43], and the Democratic Republic of Congo [36]. This can be due to the fact that the mother who visited the PNC received guidance and support care on the benefits of child immunization from health professionals.

Relative to the male household head, the odds of full childhood immunization with the female household head were higher. This finding was supported by findings in Ethiopia [15]. The finding also showed that the probability of full childhood immunization decreases as the age of the mother increases. This finding is consistent with studies in Ethiopia [21]. This can be attributed to the fact that older mothers do not have enough time to vaccinate their children due to a greater number of children and a workload for caring for their children.

## Conclusion

The full childhood immunization coverage in sub-Saharan Africa was poor with high inequalities. The finding found that there is a significant difference between SSA countries in full childhood immunization. Maternal education, maternal occupation, maternal age, marital status, father education, sex of household head, media exposure, distance to health facilities, number of ANC visits, postnatal care, place of residence, place of delivery, number of living children, wealth index, and country were significantly associated with full childhood immunization. Therefore, public health programs targeting uneducated mothers and fathers, rural mothers, poor households, and those who have not used maternal health care services to promote full childhood immunization to improve child health. By enhancing institutional delivery, antenatal care visits and maternal tetanus immunization, the government and other stakeholders should work properly to increase child immunization coverage. Furthermore, policies and programs aimed at addressing cluster variations in childhood immunization need to be formulated and their implementation must be strongly pursued.

## Data Availability

Data is available online and you can access it from www.measuredhs.com.

## Abbreviations

AIC: Akaike’s information criterion
ANC: Antenatal care
AOR: Adjusted odds ratio
CI: Confidence intervals
DIC: Deviance information criterion
EAs: Enumeration areas
DHS: Demographic and Health Survey
ICC: Intra-cluster correlation
MOR: Median odds ratio
PCV: Proportional change in variance
PNC: Postnatal care
WHO: World Health Organization
